# Comparative Analysis of Anti-receptor Binding Domain (RBD) IgG Responses to Homologous and Heterologous SARS-CoV-2 Vaccine Regimens: A Study From Bangladesh

**DOI:** 10.7759/cureus.85051

**Published:** 2025-05-29

**Authors:** Khaja Badruddza, Shahriar Habib, Sifat N Rahman, Shahadat Hossain, Farhadul H Mollah

**Affiliations:** 1 Biochemistry, Sher-E-Bangla Medical College, Barishal, BGD; 2 Microbiology, Sher-E-Bangla Medical College, Barishal, BGD; 3 Biochemistry, Gopalganj Medical College, Gopalganj, BGD; 4 Nephrology, Manikganj Medical College Hospital, Manikganj, BGD; 5 Biochemistry, Bangabandhu Sheikh Mujib Medical University, Dhaka, BGD

**Keywords:** anti-rbd igg, booster, covid-19, heterologous, homologous, immune response, sars-cov-2, vaccine

## Abstract

Background: The COVID-19 pandemic necessitated robust vaccination strategies, including booster doses to sustain immunity against SARS-CoV-2. The comparative immunogenicity of homologous (same vaccine type) versus heterologous (different vaccine types) booster regimens remains understudied, particularly in diverse settings. This study assesses anti-receptor binding domain (RBD) IgG antibody responses to these regimens in Bangladesh.

Materials and methods: A prospective quasi-experimental study was conducted at Bangabandhu Sheikh Mujib Medical University, Dhaka, Bangladesh, from March 2022 to February 2023. Seventy-three participants, selected via convenience sampling, were grouped into homologous (n=40) or heterologous (n=33) vaccine regimens based on primary and booster vaccine types. Anti-RBD IgG levels were measured pre-booster and three weeks post-booster using the SARS-CoV-2 IgG II Quant Reagent Kit (Abbott, Ireland). Demographic and clinical factors (age, sex, BMI, diabetes, and blood pressure) were evaluated. Mann-Whitney U and Kruskal-Wallis tests were used, with p≤0.05 indicating significance.

Results: Participants (mean age: 35.51 years, 79.5% male) showed higher pre-booster (median: 4499.65 vs. 1863.7 AU/mL, p<0.001) and post-booster (median: 13835.15 vs. 10423.3 AU/mL, p=0.014) anti-RBD IgG levels in the homologous group compared to the heterologous group. However, the heterologous group exhibited a greater fold increase (median: 4.6 vs. 3.65, p=0.024) of anti-RBD IgG levels. A higher proportion of participants in the homologous regimen achieved high post-booster IgG levels (>20,000 AU/mL, p=0.016). Diabetes significantly reduced antibody responses in the heterologous group (p=0.006). Hypertensive participants had significantly reduced antibody responses before (p=0.005) and after (p=0.001) the booster in the heterologous group and after (p=0.033) the booster in the homologous group. Age, sex, and BMI had no significant effect on the results.

Conclusions: Homologous regimens yield higher anti-RBD IgG levels, while heterologous regimens produce greater fold increases of anti-RBD IgG levels, indicating robust recall. Diabetes and hypertension impair responses, particularly in heterologous regimens. These findings support the need for tailored vaccination strategies to enhance immune protection.

## Introduction

The COVID-19 pandemic, caused by SARS-CoV-2, has led to unprecedented global efforts to develop and deploy effective vaccines. Multiple vaccine platforms have been authorized for emergency use worldwide, including mRNA vaccines, viral vector vaccines, protein subunit vaccines, and inactivated virus vaccines [1]. Though these vaccines have proven quite effective in preventing severe disease and death, understanding the durability and robustness of immune responses across different vaccine platforms remains crucial for optimizing vaccination strategies [2].

The receptor binding domain (RBD) of the SARS-CoV-2 spike protein contains the primary neutralizing epitopes and is a key target for protective antibodies [3]. Anti-RBD IgG antibody levels have been shown to correlate with neutralizing activity and operate as an important surrogate marker for vaccine-induced protection [4]. As immunity wanes over time following initial vaccination, booster doses have been implemented to enhance and prolong protection against emerging variants [5].

Several studies have compared the immunogenicity of different vaccine platforms, but less is known about the differential responses to booster doses across homologous versus heterologous vaccine regimens [6,7]. A homologous regimen involves receiving the same vaccine type for primary and booster doses, while a heterologous regimen involves receiving different vaccine types. Additionally, host factors including age, sex, and comorbidities may influence vaccine-induced immune responses, but these relationships are incompletely understood [7].

This study aimed to compare anti-RBD IgG antibody responses before and after booster vaccination between homologous and heterologous vaccine regimens and to identify demographic and clinical factors that may influence these responses. Understanding these patterns may inform personalized vaccination strategies and public health policies for recommending booster doses.

## Materials and methods

This research was designed as a prospective quasi-experimental study conducted at Bangabandhu Sheikh Mujib Medical University (BSMMU), Department of Biochemistry and Molecular Biology in Dhaka, Bangladesh (approval number: BSMMU/2022/7268, registration number: 3986, approval date: July 27, 2022). The research timeline spanned 12 months, from March 1, 2022, to February 28, 2023. A total of 94 individuals, who had received their second dose of the SARS-CoV-2 vaccine at least four months prior, were initially selected from various departments at BSMMU using convenience purposive sampling, based on the inclusion criteria, which required them to be healthy individuals of either gender. The study excluded individuals who tested positive for SARS-CoV-2 by reverse transcription polymerase chain reaction (RT-PCR) and those with active malignancy, severe immunosuppression, chronic liver diseases, acute infections, or pregnancy. However, 21 of them discontinued their participation. Finally, 73 participants, aged 25 to 56 years, were included in the statistical analysis.

Procedure methodology

The investigation commenced after securing approval from the university's Institutional Review Board. Each potential participant received a comprehensive explanation of the research objectives and methodologies, and informed consent was obtained for documentation from all individuals before their enrollment in the study. Age, sex, BP, BMI, and medical history were collected using a data collection sheet. A blood sample was collected for the estimation of anti-RBD IgG just before taking the booster dose. The serum was separated and preserved at -70°C. After three weeks, two blood samples were taken. One is for serum anti-RBD IgG, and the other is for RBS. Serum anti-RBD IgG before and after taking the booster dose was measured on the Alinity i Immunology Analyzer by using SARS-CoV-2 IgG II Quant Reagent Kit 06S61 (Abbott, Ireland) as directed by the manufacturer. RBS was also measured using the Alinity c Biochemical Analyzer with the Alinity c Glucose Reagent Kit (Abbott, Ireland) according to the manufacturer's instructions.

Vaccination history and grouping

Each participant's vaccination history was documented, including the type of primary series vaccines received and the timing of administration. All participants received the same vaccine or vaccine type as their first and second doses against SARS-CoV-2. The participants in this study received one of the following vaccines as their first and second doses: Oxford-AstraZeneca COVID-19 vaccine (viral vector vaccine), Pfizer-BioNTech COVID-19 vaccine (mRNA vaccine), Moderna COVID-19 vaccine (mRNA vaccine), Sinopharm COVID-19 vaccine (inactivated virus vaccine), and Sinovac COVID-19 vaccine (inactivated virus vaccine). Only the Pfizer-BioNTech COVID-19 vaccine (mRNA vaccine) was used as the booster or third dose for all participants. The study group was then split into homologous regimen and heterologous regimen groups based on whether all three doses were of the same vaccine type (mRNA) or not. A homologous booster was defined as a booster matching the platform of the first two doses or the same vaccine as the primary series. In contrast, a heterologous booster involved a different platform.

Statistical analysis

All gathered information underwent entry, cleaning, and organization before analysis using SPSS Statistics version 26.0 (IBM Corp. Released 2019. IBM SPSS Statistics for Windows, Version 26.0. Armonk, NY: IBM Corp.). For variables with normal distribution, findings were presented as means with standard deviations, while non-normally distributed variables were reported using medians and interquartile ranges. Categorical data were expressed as percentages and frequencies. Statistical analyses included the Mann-Whitney U test and the Kruskal-Wallis test, with statistical significance defined as p≤0.05. Microsoft Excel® 2019 (Microsoft Corp., Redmond, WA, USA) was used for data visualization.

## Results

**Figure 1:**
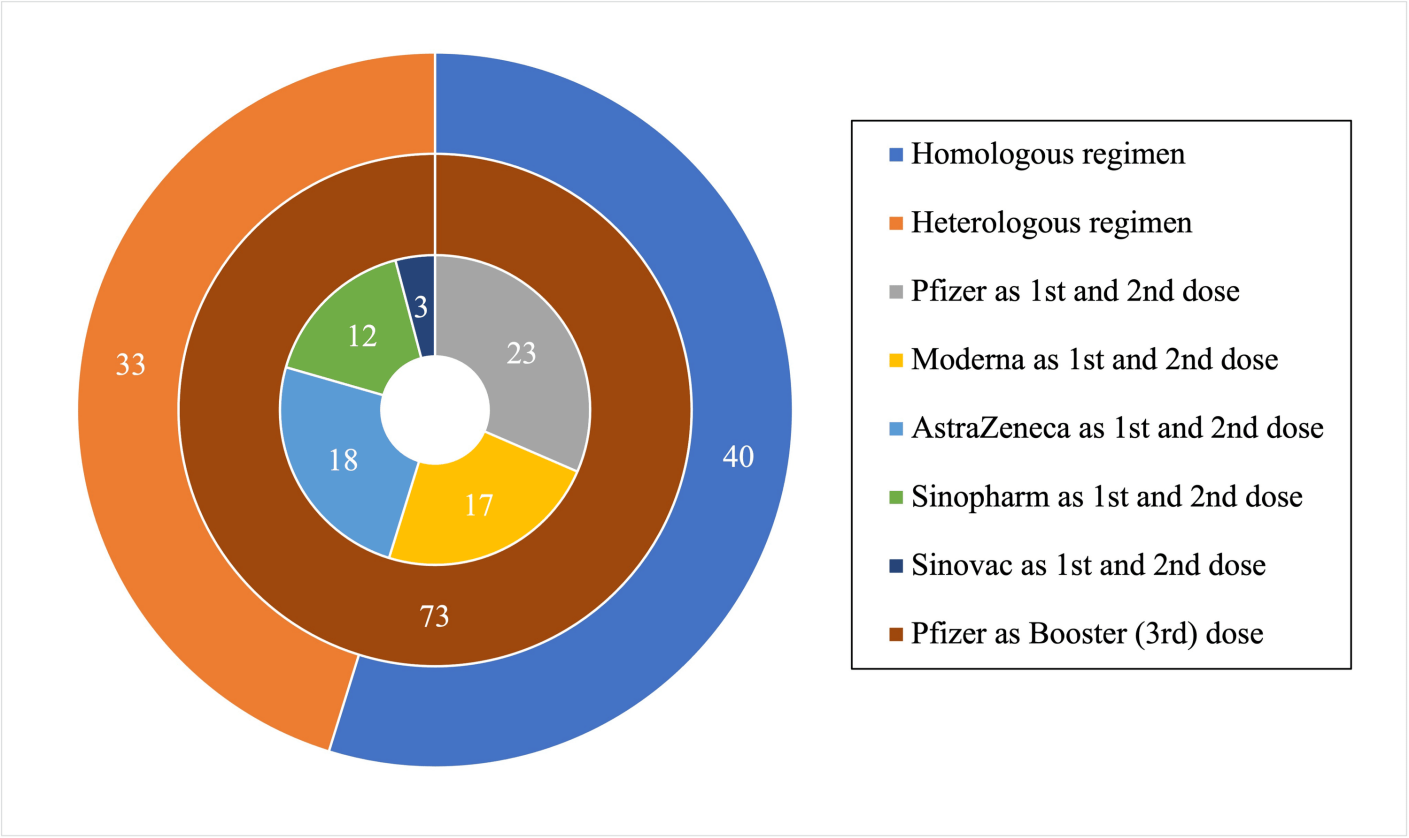
Donut chart representing the distribution of 73 participants categorized by their vaccination regimen and the types of vaccines received for their first, second, and booster (third) doses

Among the 73 participants, 58 (79.5%) were male. The age range was 25-56 years, with a mean age of 35.51 years (SD = 7.96). Based on BMI, 15 participants (20.55%) were categorized as normal, 16 (21.92%) as overweight, and 42 (57.53%) as obese. Twelve participants (16.44%) had diabetes, and 18 (24.66%) had elevated blood pressure. Most participants received the Pfizer-BioNTech COVID-19 vaccine (n=23) as their first and second doses of the COVID-19 vaccine. Oxford-AstraZeneca COVID-19 vaccine (n=18), Moderna COVID-19 vaccine (n=17), Sinopharm COVID-19 vaccine (n=12), and Sinovac COVID-19 vaccine (n=3). All 73 participants received the Pfizer-BioNTech COVID-19 vaccine as their booster or third dose, with 40 grouped into the homologous regimen and 33 into the heterologous regimen. Table 1 presents the demographic and clinical characteristics of participants by vaccine regimen, and Figure 1 illustrates the distribution of participants by vaccine regimen.

**Table 1:** Demographic and clinical characteristics by vaccine regimen SD: standard deviation, BMI: body mass index

Characteristic	Homologous regimen (n=40)	Heterologous regimen (n=33)
Gender		
Male	29 (72.5%)	29 (87.88%)
Female	11 (27.5%)	4 (12.12%)
Age (years)		
Mean ± SD	35.45 ± 8.15	35.58 ± 7.85
BMI (kg/m²)		
Mean ± SD	27.09 ± 4.47	24.98 ± 2.81
Normal	7 (17.5%)	8 (24.24%)
Overweight	9 (22.5%)	7 (21.21%)
Obese	24 (60.0%)	18 (54.55%)
Diabetes status		
Non-diabetic	35 (87.5%)	26 (78.79%)
Diabetic	5 (12.5%)	7 (21.21%)
Blood pressure		
Normotensive	30 (75%)	25 (75.76%)
Hypertensive	10 (25%)	8 (24.24%)

Comparison of anti-RBD IgG levels between vaccine regimens

Table 2 presents the summary statistics of anti-RBD IgG levels by vaccine regimen. Participants who received the homologous regimen had significantly higher pre-booster IgG levels compared to those who received the heterologous regimen (p<0.001). Similarly, post-booster IgG levels were significantly higher in the homologous group compared to the heterologous group (p=0.014). However, quite surprisingly, the fold increase in IgG levels was significantly higher in the heterologous group compared to the homologous group (p=0.024).

**Table 2:** Comparison of anti-RBD IgG levels by vaccine regimen p≤0.05. p-value determined by the Mann-Whitney U-test (two-tailed). RBD: receptor binding domain, IQR: interquartile range, IgG: immunoglobulin

Parameter	Homologous regimen (n=40)	Heterologous regimen (n=33)	U	p
Pre-booster IgG levels (AU/mL)			308	<0.001
Median	4499.65	1863.7		
IQR	2220.4-8499.85	1194.5-3196.8		
Post-booster IgG levels (AU/mL)			437	0.014
Median	13835.15	10423.3		
IQR	9077.37-27844.88	7325.3-15123.2		
Fold increase in IgG (post/pre)			455.5	0.024
Median	3.65	4.6		

Antibody response classification

Table 3 categorizes antibody responses based on post-booster IgG levels and fold increase. A higher proportion of participants in the homologous group achieved high post-booster IgG levels (>20,000 AU/mL) compared to the heterologous group (p=0.016). Conversely, a higher proportion of participants, though statistically non-significant, in the heterologous group showed strong fold increases (>8×) compared to the homologous group (p=0.16). In contrast, more participants in the homologous group showed mild (<4×) fold increases compared to the heterologous group.

**Table 3:** Antibody response classification by vaccine regimen p≤0.05. p-value determined by the Kruskal-Wallis test.

Response category	Homologous regimen (n=40)	Heterologous regimen (n=33)	Chi^2^	p
Post-booster response			8.33	0.016
High (>20,000 AU/mL)	15 (37.5%)	3 (9.1%)		
Medium (10,000-20,000 AU/mL)	14 (35%	14 (42.42%)		
Low (<10,000 AU/mL)	11 (27.5%)	16 (48.48%)		
Fold Increase			3.67	0.16
Strong (>8×)	3 (7.5%)	6 (18.18%)		
Moderate (4-8×)	13 (32.5%)	13 (39.4%)		
Mild (<4×)	24 (60%)	14 (42.42%)		

Influence of participant characteristics on antibody response

Table 4 summarizes the influence of participant characteristics on antibody responses across both vaccine regimens. No significant impact of age differences was found on both pre-booster and post-booster antibody levels. Gender differences were observed, with females exhibiting higher pre-booster antibody levels among the participants; however, this difference was not statistically significant. Both BMI category and diabetes status showed varying influences on antibody responses; however, notable differences were found in the case of diabetes status only in both pre-booster IgG (AU/mL) (p=0.05) and post-booster IgG (AU/mL) (p=0.003) levels. Significant differences were also found in the case of blood pressure status in both pre-booster IgG (AU/mL) (p=0.047) and post-booster IgG (AU/mL) (p<0.001) levels. No significant differences were found in fold increase in any category.

**Table 4:** Influence of participant characteristics on antibody response p≤0.05. p-value determined by the Mann-Whitney U-test (two-tailed) and the Kruskal-Wallis test^#^. IgG: immunoglobulin G, BMI: body mass index

Characteristic	Pre-booster IgG (AU/mL) (median)	Statistical test value	p-value	Post-booster IgG (AU/mL) (median)	Statistical test value	p-value	Fold increase (median)	Statistical test value	p-value
Age		U=631	0.786		U=646	0.916		U=643	0.89
<35 years (n=41)	2905.8			12670.7			3.9		
≥35 years (n=32)	3158.55			11972.3			4		
Gender		U=426	0.908		U=406	0.698		U=396	0.594
Male (n=58)	3015.7			11972.3			3.85		
Female (n=15)	3117.3			13165.6			4		
BMI category^#^		chi^2^=1.85	0.397		chi^2^=0.81	0.667		chi^2^=1.73	0.42
Normal/underweight (n=15)	2595.9			10423.3			4.2		
Overweight (n=16)	1802.8			11240.1			4.8		
Obese (n=42)	3194.15			13783.2			3.65		
Diabetes status		U=233	0.049		U=164	0.003		U=327	0.568
Non-diabetic (n=61)	3155.1			12713.3			4		
Diabetic (n=12)	1592.55			7092.25			3.85		
Blood pressure status		U=339	0.047		U=221	<0.001		U=376	0.13
Normotensive (n=55)	3196.8			14665.5			4.2		
Hypertensive (n=18)	1946.5			7790			3		

Subgroup analysis by vaccine regimen

Subgroup analyses of antibody responses stratified by participant characteristics within each vaccine regimen were done, where distinct patterns of influence of diabetes mellitus as a clinical factor on antibody responses between the homologous (non-significant antibody responses) and heterologous regimens (significant reduction in post-booster antibody response; p=0.006) were revealed. On the other hand, an interesting pattern was found in the case of blood pressure. While a significant reduction in only the post-booster IgG (AU/mL) levels in the homologous regimen was found (p=0.033) due to hypertension, both pre-booster IgG (AU/mL) (p=0.005) and post-booster IgG (AU/mL) (p=0.001) levels were significantly reduced in the heterologous regimens. No significant differences were found in any of the other categories. No significant differences were found in fold increase in any category.

## Discussion

Our study compared anti-RBD IgG antibody responses before and after booster vaccination across homologous and heterologous COVID-19 vaccine regimens. Both homologous and heterologous booster regimens significantly enhanced anti-RBD IgG levels, emphasizing the importance of booster doses in maintaining strong immune responses against SARS-CoV-2, a finding consistent with those of Atmar et al., Orlandi et al., and González et al. [7-9]. Our findings revealed significant differences in antibody responses between the two regimens, with the homologous regimen eliciting higher absolute antibody levels both before and after booster doses. However, the heterologous regimen showed a significantly greater fold increase in antibody titers following booster vaccination.

The higher pre-booster antibody levels observed in the homologous group suggest that this vaccine strategy may induce more durable antibody responses following the primary vaccination series. This finding aligns with previous studies showing the differential durability of antibody responses across vaccine platforms [10]. However, it's essential to note that these differences may also be influenced by factors such as the timing between primary and booster doses, which varied between the homologous and heterologous groups.

Despite starting with lower baseline antibody levels, participants who received the heterologous regimen showed a significantly higher fold increase in antibody titers following booster vaccination. This observation suggests that heterologous vaccination may induce a more robust recall response, potentially due to the engagement of different immunological mechanisms or a broader spectrum of immune stimulation. Similar findings were reported by Munro et al. [6] in the COV-BOOST trial, which demonstrated that heterologous boost regimens sometimes induced stronger antibody responses than homologous boost regimens. This phenomenon, often referred to as "mix-and-match" vaccination, has been observed in other vaccine contexts and might offer advantages in terms of breadth and diversity of immune responses [7].

Age is typically considered a significant factor influencing antibody responses, with younger participants generally exhibiting higher antibody levels, regardless of vaccine regimen. However, the findings of this study are inconsistent with previous studies, which have demonstrated age-related differences in vaccine-induced immune responses. For instance, Müller et al. [11] found that older adults exhibited lower neutralizing antibody titers following COVID-19 vaccination compared to younger adults. This age-related difference in immune response may be attributed to immunosenescence, the gradual deterioration of the immune system with age [12]. The inconsistency in this study with previous studies may have resulted from the fact that the participants in this study were significantly younger, with an overall age range of 25-56 years. In contrast, the effects of immunosenescence typically appear at a much later age [13].

The influence of gender and BMI category on antibody responses varied before and after booster vaccination, although it was not statistically significant. Among the participants, females showed higher pre- and post-booster antibody levels compared to males. Uwamino et al. [14] also demonstrated a higher antibody response among female participants compared to their male counterparts; however, due to the significant male predominance (79.5%) in the current study, the findings were not statistically significant. Research findings on the relationship between BMI and immune response to COVID-19 vaccines have been mixed. While one investigation found that obesity may not substantially affect the humoral immunogenicity of SARS-CoV-2 vaccines [15], other research indicates that individuals with very high BMI (40 kg/m² or above) show significantly reduced immune reactions to vaccination. Currently, the scientific literature doesn't provide clear conclusions regarding how moderately elevated BMI (around 25 kg/m² or higher) impacts humoral immunity following either natural SARS-CoV-2 infection or COVID-19 vaccination [16].

The diabetes status appeared to have a more pronounced impact on antibody responses in the heterologous group after the booster (p=0.006). In this study, diabetic participants exhibited significantly lower pre-booster (p=0.049) and post-booster (p=0.003) antibody levels. They also had a reduced fold increase compared to non-diabetic participants. This observation aligns with previous studies suggesting that diabetes may impair vaccine-induced immune responses [17-19], possibly due to chronic inflammation, metabolic dysregulation, or altered immune cell function [20].

Both normotensive and hypertensive individuals in this study exhibit increased anti-RBD IgG levels after receiving a booster dose of the COVID-19 vaccine, with a more pronounced effect observed in the normotensive group. Several previous studies also found similar results [21,22]. However, hypertensive participants in the heterologous group had significantly reduced antibody responses before (p=0.005) and after (p=0.001) the booster. In the homologous group, this difference was found after (p=0.033) the booster only. Hypertensive individuals displayed lower lymphocyte counts compared to normotensive individuals; as lymphocytes are crucial in the immune response to vaccination, this finding may reveal a connection between hypertension and the immune response to vaccination [22].

This study has several strengths. First, antibody levels were measured both before and after booster vaccination, allowing for the assessment of both absolute antibody levels and the magnitude of the response to boosters. Second, despite the limited sample size, a diverse range of participants were included with varying demographic and clinical characteristics, enabling the exploration of potential determinants of vaccine-induced immune responses. Ultimately, the analysis offers direct comparisons between homologous and heterologous vaccination strategies, with significant implications for public health policy.

However, this study also acknowledges some limitations. Firstly, this study only measured anti-RBD IgG antibodies, and the assessment of other components of the immune response, such as T-cell responses or neutralizing antibody activity, was not conducted. Second, some participants reached the upper detection limit of the assay (40,000 AU/mL), which may have underestimated the true magnitude of the antibody response in these individuals.

## Conclusions

This study provides valuable insights into COVID-19 booster vaccination strategies. The research also highlights how individual factors, particularly diabetes, can significantly impact vaccine responses, suggesting that vaccine strategies should be tailored to individual characteristics and circumstances. For the general population, both homologous and heterologous approaches appear effective, but with different immunological advantages. For vulnerable groups, particularly those with diabetes and hypertension, special considerations, such as prior screening for diabetes status, careful selection of a vaccine regimen based on individual health status, and monitoring and controlling blood pressure prior to vaccination, may be necessary to ensure optimal protection. This research advances our understanding of COVID-19 vaccine responses and provides evidence to support more personalized public health approaches to vaccination.
